# Унификация патоморфологического обследования пациентов с нейроэндокринными опухолями гипофиза. Спорные вопросы новой классификации

**DOI:** 10.14341/probl13376

**Published:** 2023-11-14

**Authors:** В. С. Пронин, М. Б. Анциферов, Т. М. Алексеева, Е. В. Пронин, А. М. Лапшина, Л. С. Урусова

**Affiliations:** Российская медицинская академия непрерывного профессионального образования; Эндокринологический диспансер Департамента здравоохранения города Москвы; Российская медицинская академия непрерывного профессионального образования; Эндокринологический диспансер Департамента здравоохранения города Москвы; Эндокринологический диспансер Департамента здравоохранения города Москвы; Эндокринологический диспансер Департамента здравоохранения города Москвы; Национальный медицинский исследовательский центр эндокринологии; Национальный медицинский исследовательский центр эндокринологии

**Keywords:** аденома гипофиза, нейроэндокринные опухоли гипофиза, ГипНЭО, факторы транскрипции, классификация гипофизарных нейроэндокринных опухолей ВОЗ

## Abstract

Поступательное совершенствование классификации с использованием современных аналитических методов является важнейшим инструментом для развития прецизионного и персонализированного подходов к лечению аденом гипофиза. В последние годы эндокринологи стали свидетелями эволюционных изменений, произошедших в гистопатологической идентификации новообразований гипофиза, открывающих новые возможности для изучения туморогенеза и прогнозирования биологического поведения.

В работе рассмотрены исторические аспекты поэтапного совершенствования классификации аденом гипофиза, а также новая международная классификация ВОЗ (2022 г.), согласно которой аденомы гипофиза включены в список нейроэндокринных образований (ГипНЭО), чтобы отразить биологическую агрессивность некоторых неметастатических гипофизарных аденом. Представлены клинические характеристики аденом гипофиза, а также перечень гистологических подтипов агрессивных нейроэндокринных опухолей гипофиза, отличающихся высоким потенциалом инвазивного роста, повышенным риском рецидивирования и негативным клиническим прогнозом.

Обсуждается целесообразность смены дефиниции «аденома гипофиза» на «нейроэндокринную опухоль гипофиза». Подчеркивается, что внедрение в отечественную клиническую практику унифицированного клинико-лабораторного и морфологического протокола поможет обеспечить сопоставимые сравнительные наблюдения по прогнозу заболевания и эффективности вторичной терапии, а также способствовать адекватной курации потенциально агрессивных ГипНЭО.

## Abstract (en)

The progressive improvement of the classification using modern analytical methods is an essential tool for the development of precise and personalized approaches to the treatment of pituitary adenomas. In recent years, endocrinologists have witnessed evolutionary changes that have occurred in the histopathological identification of pituitary neoplasms, revealing new possibilities for studying tumorigenesis and predicting biological behavior.

The paper considers the historical aspects of the gradual improvement of the classification of pituitary adenomas, as well as the new international 2022 WHO classification, according to which pituitary adenomas are included in the list of neuroendocrine tumors (PitNETs) to reflect the biological aggressiveness of some non-metastatic pituitary adenomas. The characteristics of pituitary adenoma are presented, as well as a list of histological subtypes of aggressive neuroendocrine tumors of the pituitary gland, marked by the main potentials for invasive growth, an increased risk of recurrence and a negative clinical prognosis.

The expediency of changing the definition of «pituitary adenoma» to «neuroendocrine tumor» is discussed. It is emphasized that the introduction of a unified clinical, laboratory and morphological protocol into national clinical practice will help provide comparable comparative studies on the prognosis of the disease and the effectiveness of secondary therapy and also contribute to adequate management of potentially aggressive PitNETs.

## Введение

Нейроэндокринные опухоли гипофиза (ГипНЭО), ранее именуемые как аденомы гипофиза, являются распространенными гетерогенными морфофункциональными образованиями с различным биологическим поведением, обусловленным гистологическим и иммунофенотипическим строением, спецификой гормональной секреции и степенью пролиферативной активности. Множественный характер морфотипов, даже в рамках одной клеточной линии, является ведущим признаком нейроэндокринных опухолей и предполагает необходимость выделения специфических предикторов для каждого гистологического подтипа и разработки персонализированного подхода к лечению [1].

Согласно последним эпидемиологическим исследованиям, распространенность опухолей гипофиза, по данным различных международных регистров, является достаточно высокой и составляет 760–1160 случаев на 1 млн жителей, тогда как заболеваемость колеблется от 39 до 82 сл./млн жителей. Средняя длительность додиагностического периода составляет около 5 лет (у 24% больных — более 10 лет). Выявляемость новообразований гипофиза за последние годы существенно увеличилась, чему способствовало широкое использование технологий нейровизуализации головного мозга и совершенствование методов гормонального анализа. По данным аутопсии и спорадического радиологического обследования, патологические образования гипофиза выявляются в 22,5–27% случаев от всех интракраниальных неоплазм, несколько уступая в численности глиомам и менингиомам. 85% из них приходится на гипофизарные аденомы. К другим селлярным образованиям относятся: краниофарингиомы (3,2%), менингиомы (0,94%), а также редко встречающиеся злокачественные опухоли и метастазы (1,2%). Среди неопухолевых процессов наблюдаются кисты кармана Ратке (2,7%), гиперплазия, абсцессы, гипофизиты (7,3%) [2–4].

Большинство аденом обнаруживаются случайно, имеют бессимптомное течение и остаются стабильными по размеру в течение длительного периода наблюдения. Такие опухоли именуются инциденталомами и составляют 12–31% от всех аденом гипофиза. Клинически манифестная опухоль наблюдается примерно у 1 из 1100 жителей и нередко проявляется характерным эндокринным симптомокомплексом и масс-эффектом, приводящим к интраселлярной и интракраниальной компрессии [5–7].

Вследствие запоздалой диагностики у 47,8% пациентов при постановке диагноза уже выявляются макроаденомы гипофиза и высокая коморбидность, что изначально ухудшает лечебный прогноз [7–9]. Спектр клинических проявлений колеблется от асимптоматических проявлений до жизнеугрожающего симптомокомплекса и включает зрительные нарушения, неврологический дефицит, гормональную дисфункцию и гипофизарную недостаточность. По данным M. Fleseriu и соавт., у 37–85% больных с макроаденомами гипофиза ко времени диагноза уже присутствуют признаки парциального гипопитуитаризма, как правило, проявляющиеся дефицитом соматотропной и гонадотропной функции. Риск прогрессирующего роста опухоли выше у больных с макроаденомами, чем с микроаденомами, и составляет 26,3% против 3,2% случаев соответственно [10].

Аденомы гипофиза образуются вследствие многоступенчатых и мультикаузальных процессов, в которых задействованы наследственная предрасположенность, специфические соматические мутации и эндокринные факторы. Важное пермиссивное значение придается непосредственному микроокружению опухоли, состоящему из неопухолевых клеток, кровеносных сосудов, внеклеточного матрикса, ферментов и цитокинов. Взаимодействие между опухолевыми клетками и компонентами микроокружения способно модулировать ангиогенез, клеточную пролиферацию и инвазивный рост и объясняет гетерогенный характер биологического поведения аденом гипофиза [8][11].

Новообразования гипофиза классифицируют в зависимости от размера, локализации, секреторной функции, типа клеток и неопластического поведения. Диагностическими характеристиками опухоли гипофиза являются ее размеры, направление экстраселлярного роста и степень инвазии. Согласно наибольшему диаметру, выделяют микроаденомы (<10 мм), макроаденомы (≥10 мм) и гигантские аденомы (≥40 мм). По данным МРТ, микроаденомы гипофиза в общей популяции выявляются в 10–38%, макроаденомы — в 0,16–0,3% случаев. МРТ с контрастным усилением является «золотым» стандартом лучевой диагностики и имеет преимущество перед КТ при выявлении образований межуточно-гипофизарной области. Чувствительность МРТ при визуализации микроаденом составляет 82,6% по сравнению 42,1% при использовании КТ [6].

Инвазию кавернозного и клиновидного синусов (0–4-й степени) оценивают с использованием классификации E. Knosp и J. Hardy [8]. Большинство аденом являются относительно доброкачественными образованиями с малым объемом и медленным интраселлярным развитием. Их можно радикально удалить или стойко контролировать с помощью традиционных методов лечения [10][11]. Что же касается макро- и гигантских аденом, то они, как правило, имеют инвазивный характер и проявляются угрожающими неврологическими и сосудистыми осложнениями внутричерепной компрессии. Масс-эффект характеризуется парциальным гипопитуитаризмом, постоянной головной болью, головокружением и зрительными нарушениями, что обусловлено анатомическим расположением гипофиза вблизи критически важных нервных и сосудистых структур головного мозга. Поэтому при больших размерах аденомы и ее экстраселлярном распространении больному, как правило, требуется оперативная коррекция для устранения или профилактики компрессионного синдрома. Однако, в связи с запоздалой диагностикой, примерно в 50% случаев не удается достичь послеоперационной ремиссии, что требует дополнительного лечения [2][9][12][13].

Гормонально активные аденомы гипофиза оказывают длительное разноплановое эндокринное воздействие на организм, приводящее к высокой коморбидности и сокращению качества и продолжительности жизни. Диапазон клинических признаков колеблется от незначимых симптомов до тяжелых метаболических нарушений. По данным разных национальных когорт, среди клинически активных гипофизарных опухолей 32–66% секретируют пролактин (ПРЛ), около 10–16% — гормон роста (ГР), примерно 2–6% — адренокортикотропный гормон (АКТГ) и менее 1% — ТТГ. Функционально неактивные опухоли представлены в 36–54% случаев [1][14]. Большинство аденом имеет спорадический характер, и только незначительная их часть является проявлением врожденных генетических нарушений, включая синдромы МЭН 1 и 4 типов, комплекс Карни, синдром МакКьюна-Олбрайта, семейную изолированную гипофизарную аденому, Х-сцепленный акрогигантизм [15–18].

Несмотря на доброкачественный морфологический статус аденомы, «не причиняющий проблем для здоровья пациента», примерно у 31–35% больных наблюдаются «клинически агрессивные» гистотипы, характеризующиеся активным инвазивным ростом в окружающие анатомические структуры, рецидивирующим послеоперационным течением и высоким риском малигнизации. Данные аденомы отличаются рефрактерностью к лекарственной и лучевой терапии и требуют повторного использования радикальных методов лечения, нередко с исходом в гипофизарную и вазоцеребральную недостаточность, что негативно влияет на качество жизни и срок дожития [12][19][20].

Следует подчеркнуть, что «агрессивные» варианты аденом, исходящие из различных трофных клеток аденогипофиза, занимают клинически тревожную нишу между типичными аденомами и карциномами и являются наиболее проблемными образованиями, не укладывающимися в классическую дефиницию аденом (доброкачественные эпителиальные опухоли), имеющих «безоблачный» лечебный прогноз. Именно из этих клинически агрессивных гистологических подтипов в последующем могут образоваться гипофизарные карциномы с системным метастазированием и быстрым фатальным исходом. Поэтому, с учетом наличия множественных патоморфологических вариантов гипофизарных неоплазий с возможностью непредсказуемого биологического поведения и высоким риском малигнизации, в последней классификации ВОЗ гипофизарные аденомы были переименованы в гипофизарные нейроэндокринные опухоли (ГипНЭО), чтобы подчеркнуть практическую важность скорейшей диагностики заболевания, дифференцированного подхода к лечению и пожизненного динамического контроля.

Что касается гипофизарных карцином, то они встречаются в 0,1–0,5% случаев, образуются из любых типов клеток (чаще из кортикотропных или пролактотропных) и могут иметь различную функциональную активность. Ускоренный рост опухолевой массы способствует интраселлярной и интракраниальной компрессии, избыточной гормональной секреции, цереброспинальному и системному метастазированию. Характерной чертой является продолженный резидуальный рост и рецидивирование, несмотря на активное мультимодальное лечение. При этом отсутствие или наличие метастазов является, к сожалению, единственным отличительным свойством между «агрессивными» аденомами и гипофизарными карциномами, что требует особого внимания к аденомам с необычным клиническим течением [21][22].

## Хронологические этапы классификаций аденом гипофиза

Международные классификации неоплазий гипофиза представляют собой динамично меняющуюся область совокупных знаний, отражающую достижения в клеточной биологии и более глубокое понимание патогенетических механизмов туморогенеза. Многообразие существующих ультраструктурных гистологических вариантов аденом гипофиза, даже в пределах одного клинико-лабораторного симптомокомплекса, усложняет постановку клинического диагноза и затрудняет выработку адекватной лечебной концепции. Поэтому на примере поступательного совершенствования классификации аденом (опухолей) гипофиза можно проследить положительную динамику аналитических методов диагностического поиска и все возрастающую роль квалифицированного патоморфологического диагноза в стратификации и клиническом прогнозировании новообразований хиазмально-селлярной области. В итоге это является важнейшим инструментом для реализации прецизионного и персонализированного подходов к лечению нейроэндокринных нарушений.

Со времени первых клинических описаний международная классификация аденом гипофиза претерпела 5 редакций, каждая из которых отражала текущий уровень знаний и новые возможности морфофункционального анализа, что в итоге сформировало ее диагностическую и прогностическую направленность. Первая «тинкториальная» классификация аденом гипофиза была создана в конце XIX века и построена на особенностях окрашивания опухолевых клеток специфическими гистологическими красителями (гематоксилином и эозином), предложенными немецким патологом Карлом Бенде. Исходя из тинкториальных характеристик цитоплазмы клеток после окрашивания Шонеман в 1892 г. предложил разделить образования гипофиза на хромофильные [ацидофильные (эозинофильные) и базофильные] и хромофобные типы. Далее было установлено, что ацидофильные (эозинофильные) опухоли способны секретировать ГР или ПРЛ, базофильные — АКТГ, ТТГ, ЛГ и ФСГ, тогда как наиболее часто встречающиеся хромофобные аденомы были полностью лишены гормональной активности. Термин «аденома гипофиза» для обозначения доброкачественных новообразований был впервые предложен Г. Кушингом в 1912 г. [23][24].

Таким образом, первая диагностическая концепция строилась на выделении определенного клинического симптомокомплекса, априори указывающего на присутствие соответствующей по диагностическому окрашиванию аденомы. Классической иллюстрацией является высказывание И.А. Бейлина (1938 г.), отметившего, «что акромегалия есть именно та болезнь, когда при жизни и при одном внешнем осмотре можно поставить не только топический, но и гистологический диагноз» [25].

«Тинкториальная» классификация аденом гипофиза с различными дополнениями просуществовала практически до 1980-х гг. За этот период накопились факты, указывающие на неполное соответствие между морфологическими и клиническими данными. Был выделен ряд характеристик, отличающих некоторые типы аденом от обычных доброкачественных опухолей, включая склонность к кровоизлияниям и некрозу, частую инвазию в близлежащие структуры, рефрактерность к лечению и риск метастазирования. Обнаружено, что не все ацидофильные опухоли продуцируют ГР и не все ГР-продуцирующие опухоли состоят из ацидофильных клеток. Дополнительно было обращено внимание на разную интенсивность окрашивания, коррелирующую с нюансами клинического течения. Некоторые базофильные опухоли проявлялись клинически не болезнью Кушинга, а акромегалией. Более того, оказалось, что примерно в 50% случаев хромофобные опухоли являются гормонально активными, секретируя в различных вариациях тропные гормоны. Поэтому, с позиции новых знаний, «тинкториальная» классификация оказалась слишком упрощенной, не отражающей существующего морфофункционального многообразия гипофизарных неоплазий.

Внедрение в клиническую практику иммуноэлектронной микроскопии позволило глубже понять некоторые аспекты цитопатологии гипофиза и обеспечить более точную морфологическую диагностику заболевания с выделением смешанных и гормонально неактивных опухолей. Полученные данные послужили основой для разработки 2-й, «ультраструктурной» классификации, дополнительно разделяющей аденомы гипофиза на отдельные внутригрупповые гистологические подтипы, коррелирующие с цитоархитектоникой аденоматозных клеток и эндокринной активностью. Среди гипофизарных аденом были выделены аденомы с большим количеством плотно расположенных секреторных гранул (так называемые плотногранулированные опухоли), аденомы с меньшим количеством и редко расположенными секреторными гранулами (редкогранулированные опухоли), бицеллюлярные аденомы, а также низкодифференцированные гистотипы, которые, несмотря на схожую гормональную направленность, различались клиническим поведением, выраженностью инвазивного роста и чувствительностью к аналогам соматостатина 1-й генерации (АС1) [26–28].

Выявленная неоднозначность клинических и морфологических проявлений аденом гипофиза, а также появление новых гистологических подтипов, часть из которых отличалась агрессивным поведением и рефрактерностью к фармакотерапии, требовали дополнительной систематизации. 3-я морфологическая классификация аденом гипофиза ВОЗ от 2004 г., которую условно можно обозначить как «гистопатологическая», была основана на комбинации гистологических, иммуногистохимических, электронно-микроскопических и клинических данных. Предлагалось стратифицировать аденомы путем комбинации двух главных гистопатологических признаков: присутствия в опухолевых клетках гормональной активности (с использованием иммуногистохимического окрашивания) и определения ультраструктурных биологических маркеров с оценкой рецепторного и пролиферативного фенотипов опухолевой ткани. Аденома гипофиза рассматривалась как клональная неопластическая пролиферация гормон-продуцирующих клеток передней доли гипофиза. Все аденомы были подразделены на 3 категории (типичная аденома, атипичная аденома и гипофизарная карцинома). Типичные гормонально активные аденомы были разделены на несколько подтипов: АКТГ-, ГР-, ПРЛ-, ТТГ- и ЛГ/ФСГ-секретирующие аденомы. Дефиниция «атипичной» аденомы характеризовалась агрессивным течением, повышенным количеством митозов, высоким пролиферативным индексом Ki-67 (>3%) и наличием позитивной иммунореактивности белка р53. Гипофизарную карциному отличал ускоренный клеточный рост (индекс Ki-67 >10%) и наличие цереброспинальных и/или системных метастазов [29][30].

Это была первая попытка идентифицировать гипофизарные опухоли, отличающиеся от доброкачественных аденом экстенсивным ростом и потенциально злокачественной трансформацией. Было отмечено, что «атипичные» аденомы, выявляемые в 2,7–15% случаев, имеют сходные морфологические и гистологические черты с гипофизарными карциномами, характеризующимися ускоренным ростом, отдаленным метастазированием и быстрым летальным исходом [23][31].

В 2017 г. была опубликована 4-я, «гистогенетическая», редакция классификации ВОЗ эндокринных опухолей, в которой формальное разделение моноклональных аденом аденогипофиза по гормональному признаку было дополнено стратификацией по эмбриональному происхождению. Как известно, формирование гипофиза происходит на 5–13 неделях внутриутробного развития. Передняя доля гипофиза развивается из оральной эктодермы путем эпителиального выпячивания дорсальной стенки ротовой бухты в виде пальцевидного выроста (кармана Ратке), направляющегося к основанию головного мозга, в области 3-го желудочка, где встречается с будущей задней долей гипофиза, которая развивается из отростка воронки промежуточного мозга. Дальнейшая дифференцировка стволовых клеток кармана Ратке и формирование рецепторного аппарата питуицитов в процессе эмбрионального и постнатального развития зависит от модулирующего воздействия факторов транскрипции (ФТ), обеспечивающих различные направления типовой и видовой специализации секреторных клеток из общего источника. [Среди первичных ФТ выделяют: PIT1 (pituitary-specific POU-class homeodomain transcription factor 1), TPIT (T-box family member TBX19) и SF1 (steroidogenic factor 1)] [20].

В итоге эмбрионального развития из общего пула клеток аденогипофиза образовалось несколько дифференцированных линий (семейств), стратифицированных по ведущему фактору транскрипции. Так, к PIT1-зависимому семейству принадлежат лактотрофы, соматотрофы и тиреотрофы. При этом ФТ PIT1 функционально связан с вторичными ФТ: ERα (estrogen receptor α) и GATA2 (member of GATA family of zinc-finger transcriptional regulatory proteins), необходимыми для дальнейшей дифференцировки лактотрофов и тиреотрофов. ФТ TPIT обеспечивает транскрипцию проопиомеланокортиновой линии в дифференцированные кортикотрофы. Стероидогенный фактор (SF1) через GATA2 регулирует дифференцировку гонадотрофов [32][33] (рис. 1, табл. 1).

Как следует из рис. 1, самым множественным является PIT1-зависимое семейство, объединяющее клетки с незавершенной (стволовые соматотрофы, маммосоматотрофы) и завершенной (соматотрофы, лактотрофы, тиреотрофы) терминальной дифференцировкой. В новом варианте были закреплены ультраструктурные и иммуногистохимические особенности клеток сходной гормональной направленности, позволяющие проводить более тонкую дифференциальную диагностику между гистологическими подтипами, имеющими различное клиническое течение [35].

**Figure:**
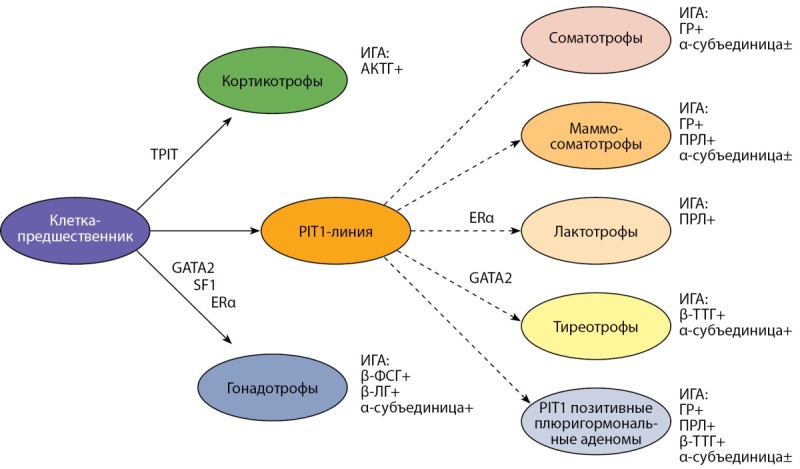
Рисунок 1. Схема видовой дифференцировки клеток аденогипофиза.

В таблице 1 представлены клеточные линии аденогипофиза, первичные и вторичные факторы транскрипции, обеспечивающие направленную видовую дифференцировку и поддержание клеточной специализации, а также конечные виды специализированных клеток с завершенной терминальной дифференцировкой.

**Table:** Таблица 1. Аденогипофизарные клеточные линии

Клеточные линии	Ведущие факторы транскрипции и другие кофакторы	Аденогипофизарные клетки
Ацидофильная линия	PIT1	Соматотрофы
	PIT1, ERα	Лактотрофы
	PIT1, GATA2	Тиреотрофы
Кортикотрофная линия	TPIT	Кортикотрофы
Гонадотрофная линия	SF1, GATA2, ERα	Гонадотрофы

Таким образом, принципиальным отличием новой редакции была первичная стратификация аденом гипофиза согласно принадлежности к определенной аденогипофизарной клеточной линии с последующим определением гистологических вариантов с учетом гормонального контента и наличия специфических гистологических и иммуногистохимических признаков. При этом в качестве ведущего инструмента стал использоваться иммуногистохимический анализ (ИГА).

В классификации ВОЗ от 2017 г. впервые было указано, что сам гистотип (или подтип) аденомы может иметь прогностическое значение, поскольку обладает своеобразным сценарием биологического поведения, требующим учета при лечебной коррекции. Термин «атипичная» аденома был отменен из-за отсутствия доказательных предикторов ее агрессивной принадлежности. Вместо этого было предложено разделять аденомы гипофиза на аденомы «низкого» и «высокого» злокачественного риска, основываясь на их пролиферативной активности. Так были выделены морфотипы аденом высокого злокачественного риска, априори имеющие агрессивное течение: редкогранулированная соматотрофная аденома, лактотрофная аденома у мужчин, аденома из Круковских клеток, «молчащая» кортикотрофная опухоль и плюригормональная PIT1-позитивная аденома. Эти гистологические типы (подтипы) аденом отличаются ускоренным ростом, тенденцией к рецидивированию и прогрессированию, а также резистентностью к медикаментозному и/или радиологическому лечению [1][35–37].

Следует отметить, что выявление ФТ с помощью ИГА значительно расширяло диагностические возможности морфологического анализа, но не исключало необходимости определения особенностей гормональной экспрессии, имеющей решающее значение для суждения о секреторной активности опухоли. Дополнительное типирование с использованием данных электронной микроскопии было исключено из последних рекомендаций, поскольку данная методика редко используется в рутинной практике. Вместо этого была рекомендована оценка маркеров клеточной пролиферации, инвазии и гистологических подтипов, необходимых для выявления опухолей с агрессивным потенциалом. Нуль-клеточные аденомы были более четко определены как опухоли, в которых не наблюдалось какой-либо дифференциации по типу клеток, а также иммуноэкспрессии гормонов и факторов транскрипции. Что касается диагностических критериев карциномы гипофиза, то они остались неизменными и формулируются как опухоли из клеток аденогипофиза, способные к краниоспинальному или системному метастазированию [38][39].

Одним из знаковых событий последних лет стало включение аденом гипофиза в список нейроэндокринных неоплазий. В 5-й классификации эндокринных опухолей и опухолей центральной нервной системы ВОЗ от 2022 г., которую условно можно обозначить как «онкологическая», была завершена работа по биологической идентификации гистологических подтипов аденом гипофиза с внесением их в список нейроэндокринных опухолей в качестве отдельного семейства «ГипНЭО». Одновременно в данной редакции была пересмотрена и изменена номенклатура нейроэндокринных опухолей всех локализаций тела с созданием объединенной классификации и утверждением стандартного протокола верификации морфологического диагноза [40][41] (рис. 2).

**Figure:**
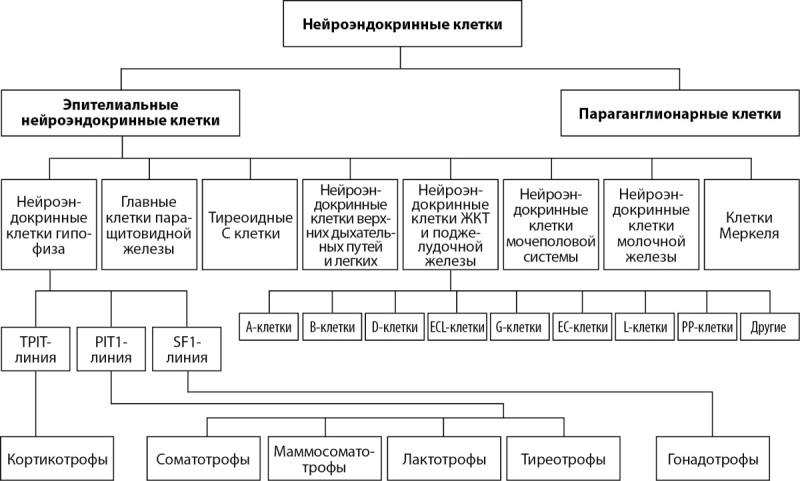
Рисунок 2. Нейроэндокринные клетки и органы, входящие в APUD-систему [41].

Новая редакция классификации ВОЗ от 2022 г. была принята согласно предложению экспертов Европейского клуба гипофизарной патологии [European Pituitary Pathology Group (EPPG)] и принципиально изменила патоморфологический статус аденомы гипофиза, закрепив новую диагностическую дефиницию — «нейроэндокринная опухоль гипофиза» (ГипНЭО). Основанием для этого решения стали, во-первых, эмбриональная принадлежность аденогипофизарных клеток к нейроэндокринному семейству, во-вторых, высокий риск малигнизации, метаболических и системных нарушений, компрессионного и/или инвазивного воздействия аденом гипофиза на окружающие ткани и, в-третьих, рефрактерность к медикаментозной терапии, не препятствующей продолженному росту новообразования.

«Онкологическая» классификация четко отличает опухоли передней доли (аденогипофизарные) от опухолей задней доли (нейрогипофизарные) и гипоталамуса. Основные типы и подтипы ГипНЭО, определяемые линиями PIT1, TPIT и SF1, имеют четкие морфологические, молекулярные и клинические различия. В отличие от классификации 2017 г., версия ВОЗ от 2022 г. обновила некоторые диагностические концепции:

1) маммосоматотрофные, ацидофильные опухоли стволовых клеток и смешанные соматотрофные и лактотрофные опухоли теперь представляют собой самостоятельные образования линии PIT1;

2) PIT1-положительная плюригормональная опухоль подразделена на незрелую (ранее именовалась «молчащая» опухоль 3-го подтипа) и зрелую плюригормональную опухоли PIT1-линии;

3) «Нуль-клеточная опухоль» является диагнозом исключения и зарезервирована для ГипНЭО без признаков дифференцировки аденогипофизарной линии. Вместо предыдущего термина «карцинома гипофиза» предложен термин «метастатическая гипофизарная нейроэндокринная опухоль» («метастатический ГипНЭО»). Онкологический код Международной классификации болезней был пересмотрен с «0» для доброкачественных опухолей на «3» для первичных злокачественных опухолей, а также для нейроэндокринных опухолей других органов [13][42] (табл. 2).

**Table:** Таблица 2. Классификация гипофизарных нейроэндокринных опухолей ГипНЭО ВОЗ от 2022 г. [42]

ГипНЭО	Факторы транскрипции	Гормоны	Низкомолекулярный цитокератин (CAM5.2)	Подтипы
PIT1-линия ГипНЭО
Соматотрофные опухоли	PIT1	ГР, α-субъединица (с/е)	перинуклеарная локация	плотногранулированная соматотрофная опухоль
PIT1	ГР	фиброзные тельца (>70%)	редкогранулированная соматотрофная опухоль
Лактотрофные опухоли	PIT1, ERα	ПРЛ (парануклеарно)	слабая или негативная экспрессия	редкогранулированная соматотрофная опухоль
PIT1, ERα	ПРЛ (диффузное цитоплазматическое	слабая или негативная экспрессия	плотногранулированная лактотрофная опухоль
Маммосоматотрофная опухоль	PIT1, ERα	ГР (предоминирует), ПРЛ, α-субъединица	перинуклеарная экспрессия	
Тиреотрофная опухоль	PIT1, GATA3	α-с/е, β-ТТГ	слабая или негативная экспрессия	
Зрелая плюригормональная опухоль PIT1-линии	PIT1, ERα, GATA3	ГР (предоминирует), ПРЛ, α-с/е, β-ТТГ	перинуклеарная локация	
Незрелая опухоль PIT1-линии	PIT1, ERα, GATA3	фокальная/вариабельная экспрессия гормонов PIT1-линии (ГР, ПРЛ, β-ТТГ, α-с/е)	фокальная/вариабельная экспрессия	
Ацидофильная опухоль из стволовых клеток	PIT1, ERα,	ПРЛ (предоминирует), ГР (фокальная/вариабельная)		
Смешанная соматотрофная и лактотрофная опухоль	PIT1, ERα	соматотрофный компонент (ГР±α-с/е)	в зависимости от типа/подтипа опухолей	комбинация любых соматотрофных и лактотрофных подтипов
лактотрофный компонент: ПРЛ (диффузное или парануклеарное распределение в зависимости от подтипа)
TPIT-линия ГипНЭО
Кортикотрофные опухоли	TPIT	АКТГ	выраженная экспрессия	плотногранулированная кортикотрофная опухоль
вариабельная экспрессия	редкогранулированная кортикотрофная опухоль
кольцеобразное цитоплазматическое перинуклеарное	опухоль из Круковских клеток
SF1-линия ГипНЭО
Гонадотрофная опухоль	SF1, ERα, GATA3	α-c/е, β-ФСГ, β-ЛГ, или негативная	вариабельная или негативная экспрессия	
ГипНЭО с неуточненной линейной дифференцировкой
Плюригормональная опухоль	множественные комбинации	множественные комбинации	вариабельная экспрессия	
Нуль-клеточная опухоль	нет	нет	вариабельная экспрессия	

Предлагаемая в новой классификации смена дефиниций «опухоль» вместо «аденома» отражает широкий спектр клинического поведения новообразований гипофиза от вялотекущего присутствия до выраженных метаболических и компрессионных поражений, характеризуя, таким образом, агрессивный характер течения заболевания без однозначных патогистологических признаков злокачественности. Термин «опухоль» является более общим понятием, объединяющим доброкачественные и злокачественные неоплазии, тогда как дефиниция «аденома» относится к сугубо доброкачественному новообразованию эпителиального происхождения. Предлагаемая группой экспертов ВОЗ смена терминов обозначала констатацию непредсказуемости и потенциальной злокачественности некоторых аденом гипофиза. Исходя из принадлежности клеток гипофиза к нейроэндокринному семейству, было рекомендовано использовать термин «ГипНЭО», отражающий гетерогенность гистологических подтипов гипофизарных опухолей и возможность различных клинических сценариев [43].

Данная терминология идентифицирует новообразования гипофиза на основании молекулярно-биологических особенностей гипофизарных клеток как представителей диффузной нейроэндокринной системы (или APUD-системы), клетки которой обладают способностью поглощать аминокислоты-предшественницы и производить из них активные амины и/или низкомолекулярные пептиды с помощью реакции декарбоксилирования (Amine Precursor Uptake and Decarboxylation) [44]. В настоящее время выделяют два подсемейства нейроэндокринных клеток: А — эпителиальные нейроэндокринные клетки, происходящие главным образом из эмбриональной энтодермы, и Б — неэпителиальные нейроэндокринные клетки (параганглии), происходящие из эмбриональной нейроэктодермы. Пул эпителиальных нейроэндокринных клеток объединяет аденогипофизарные клетки, главные клетки паращитовидной железы, парафолликулярные С-клетки щитовидной железы, островковые клетки поджелудочной железы и клетки так называемой рассеянной нейроэндокринной системы, которые населяют слизистую оболочку дыхательного, желудочно-кишечного и мочеполового трактов [45][46] (рис. 3).

**Figure:**
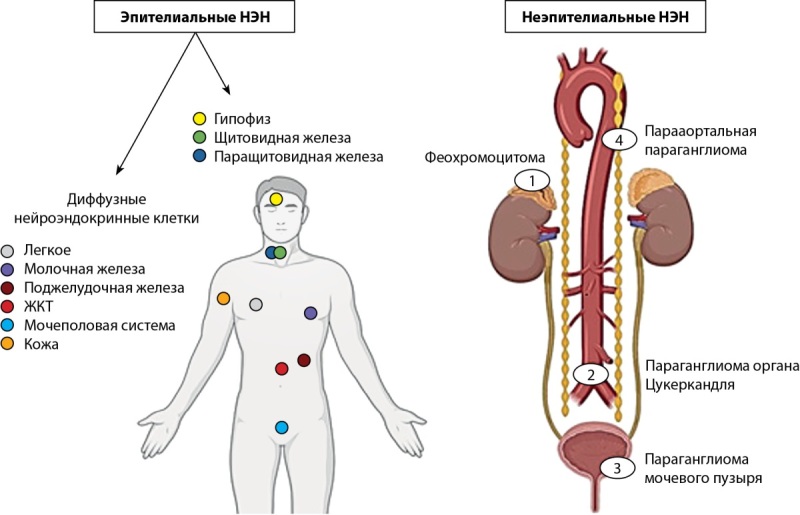
Рисунок 3. APUD-система. Расположение эпителиальных и неэпителиальных нейроэндокринных клеток [45].

Согласно новой классификации, клетки аденогипофиза представляют собой отдельное семейство нейроэндокринных клеток, принадлежность которых к APUD-системе проявляется в способности экспрессировать ряд характерных белков, включая молекулы адгезии нервных клеток, нейронспецифическую энолазу, синаптофизин, хромогранины, кератины и фактор транскрипции, ассоциированный с инсулиномным белком-1.

Нейроэндокринная опухоль была впервые описана в 1907 г. З. Оберндорфером, который ввел термин «карциноид» («ракоподобный») для описания опухолей, обнаруженных в тонкой кишке [47]. Позднее было доказано, что нейроэндокринные неоплазии (НЭН) [neuroendocrine neoplasms (NENs)] возникают практически во всех органах, имеющих нейроэндокринные клетки, и, согласно гистологическим и иммунофенотипическим критериям, подразделяются на высокодифференцированные неоплазмы, именуемые нейроэндокринными опухолями (НЭО) [neuroendocrine tumors (NETs)] и низкодифференцированные неоплазмы, определяемые как нейроэндокринные карциномы (НЭК) [neuroendocrine carcinomas (NECs)]. В свою очередь НЭО представляют собой гетерогенную группу эпителиальных и неэпителиальных новообразований, биологическое поведение которых зависит от места происхождения и степени пролиферации опухоли. Независимо от видовой принадлежности, эти опухоли различаются функциональным статусом, степенью агрессивности и отдаленным лечебным прогнозом. Термин «метастатическая гипофизарная нейроэндокринная опухоль» предложен для обозначения агрессивных низкодифференцированных нейроэндокринных новообразований высокой степени злокачественности. При этом подчеркивается важность пролиферативного индекса Ki-67 как одного из инструментов классификации и градации [48–50].

## Стратификация нейроэндокринных опухолей

Внедрение стандартного протокола морфологического анализа позволяет определить типовую и видовую принадлежность опухолевого образования, его пролиферативный потенциал и особенности рецепторной экспрессии, что в итоге предоставляет возможность оценить отдаленный прогноз развития заболевания и наметить персонализированную программу его лечения. По мнению создателей, классификация ВОЗ от 2022 г. требует детального гистологического подтипирования опухолей с учетом клеточной линии аденогипофиза, типа клеток и других характеристик. Особый акцент сделан на роли диагностических иммуногистохимических биомаркеров для подтверждения нейроэндокринной природы новообразования. Одновременно рекомендовано рутинное определение факторов транскрипции гипофиза (PIT1, TPIT, SF1, GATA3 и ERα) наряду с гормонами (ГР, ПРЛ, β-ТТГ, β-ФСГ, β-ЛГ, АКТГ и α-субъединица), кератином (рекомендуемый клон CAM5.2) и Ki-67 для точного определения типа новообразований, необходимого для предсказания клинических, патологических и молекулярно-патогенетических особенностей отдельных гистологических подтипов (рис. 4).

**Figure:**
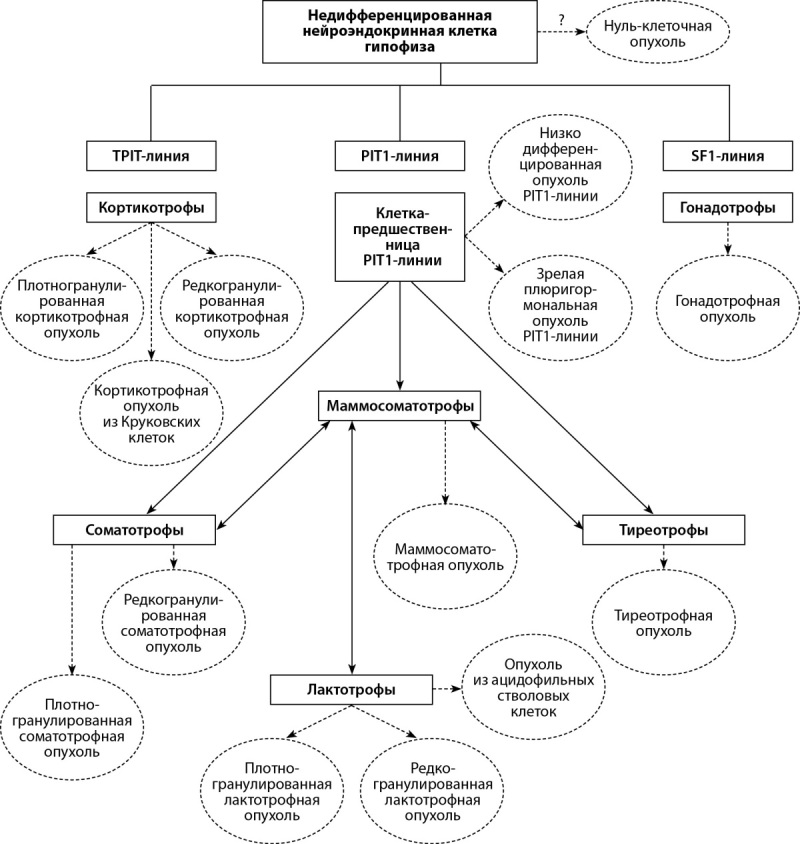
Рисунок 4. Современные типы и гистологические подтипы ГипНЭО [41].

В условиях множественности гистологических подтипов использование рутинного подтипирования играет важнейшую роль в идентификации новообразования и прогнозирования его клинического поведения. Например, в список ГР-продуцирующих аденом включены 7 патоморфологических вариантов согласно присутствию специфических гистологических и иммуногистохимических маркеров: плотногранулированные, редкогранулированные, маммосоматотрофные, сомато-лактотрофные аденомы, а также зрелые плюригормональные и незрелые аденомы PIT1-линии и аденомы из ацидофильных стволовых клеток, характеризующиеся небольшой секреторной активностью, но ускоренным и инвазивным ростом. Среди «чистых» лактотрофных аденом выделяют патоморфологические варианты, состоящие из плотно- и редкогранулированных клеток. В свою очередь TPIT-семейство объединяет 3 гистологических подтипа аденом, состоящих из плотногранулированных базофильных клеток, редкогранулированных хромофобных клеток и гиалинизированных Круковских клеток. Если раньше было известно только о трех морфологических вариантах аденом гипофиза (эозинофильные, базофильные и хромофобные), то в настоящее время насчитывается 18 гистологических типов/подтипов, что существенно повышает значимость точного морфологического анализа для дифференциальной диагностики [37][41][50].

Оценка клеточной дифференцировки при эндокринных новообразованиях включает не только описание клеточной структуры и экспрессию специфических ФТ, регулирующих цитогенез, но также идентификацию гормонов и/или ферментов, принимающих участие в гормональном синтезе. Важность определения функциональной активности опухоли связана с тем, что гормоны служат своеобразными биомаркерами для клинического наблюдения, сигнализируя о персистенции, продолженном росте или рецидиве гормонально-активного заболевания. Прогрессирующее снижение гормональной секреции может быть признаком клеточной дедифференцировки, сопряженной с более агрессивным течением [51].

Независимо от поступательного совершенствования методов биохимического и радиологического анализа при диагностике ГипНЭО, гистологическая диагностика остается «золотым» стандартом для выявления опухолевых характеристик, необходимых для принятия лечебного решения. В эру все возрастающей потребности в точной диагностике с использованием минимального количества биопсийного материала ИГА стал незаменимым вспомогательным инструментом для цитологической классификации этих опухолей. С учетом синдромального характера известных нозологических форм гиперфункции гипофиза (с включением эктопических и метастатических новообразований) послеоперационный ИГА является обязательной опцией для линейной и внутригрупповой классификации опухолей гипофиза, позволяющей обеспечить персонализированный подход к лечению заболевания [46]. Протокол стратификации нейроэндокринных опухолей ГипНЭО предполагает:

1) подтверждение нейроэндокринной дифференцировки опухоли.

К наиболее надежным иммуногистохимическим маркерам нейроэндокринных клеток относятся синаптофизин, хромогранин А и фактор транскрипции, ассоциированный с белком-1 инсулиномы. Синаптофизин часто рассматривается как наиболее чувствительный маркер при диагностике хорошо дифференцированных НЭО. Что касается хромогранина А, то уровень его экспрессии может варьировать в зависимости от степени дифференцировки опухоли. Очаговое, слабое или даже отсутствующее окрашивание хромогранином А может быть отмечено при низкодифференцированных опухолях. В ряде случаев рекомендуется одновременное применение хромогранина А и синаптофизина для точного подтверждения (или исключения) нейроэндокринной дифференцировки;

2) исследование маркеров эпителиальной дифференцировки.

С этой целью используется панель эпителиальных биомаркеров на цитокератины (AE1/AE3, CAM5.2, СК18) для подтверждения эпителиальной природы и исключения кератин-негативной нейроэндокринной опухоли (потенциальной параганглиомы);

3) определение маркеров опухолевой пролиферации (Ki-67/MIB-1), отражающих инвазивный и метастатический потенциал.

В нейроэндокринных опухолях негипофизарной локализации (например, поджелудочной железы или легкого) опухолевая пролиферация оценивается согласно выраженности пролиферативных признаков, ранжированных по системе G1-G3. Ранг G1 ставится при отсутствии некроза и наличии <2 митозов на 2 мм² и при величине пролиферативного индекса Ki-67 <20%. Для ранга G2 характерно наличие некроза или 2–10 митозов на 2 мм² и Ki-67 <20%. Ранг G3 устанавливается при присутствии >10 митозов на 2 мм² или величины Ki-67 >20%, а также отсутствии низкодифференцированной цитоморфологии. Нейроэндокринные карциномы регистрируются при наличии >10 митозов на 2 мм² и величине Ki-67 >55% и подразделяются на мелкоклеточные и крупноклеточные нейроэндокринные карциномы [46][48]. (Однако для опухолей аденогипофиза подобная система градации в настоящее время не разработана.);

4) идентификация видового клеточного происхождения опухолевой ткани.

В сочетании с клиническими и цитоморфологическими данными панели маркеров факторов транскрипции и гормонов обеспечивают высокую чувствительность и специфичность для идентификации места происхождения. При подозрении на опухоль гипофиза (или с целью определения источника метастатического поражения неизвестной первичной локализации) положительный результат на факторы транскрипции (PIT1, TPIT и SF1) будет полезен для подтверждения происхождения опухоли из клеток аденогипофиза;

5) определение биомаркеров, имеющих потенциальное прогностическое или терапевтическое значение.

Речь идет о выявлении выраженности экспрессии 2-го и 5-го подтипов соматостатиновых рецепторов, характере гормональной активности, присутствии дополнительных субъединиц, локации цитокератинов, содержании Е-кадгерина, β-катенина и других антител в образцах ткани удаленных опухолей гипофиза;

6) генетическое консультирование.

Всем молодым пациентам и членам их семей рекомендована консультация врача-генетика с рассмотрением вопроса о проведении генетического обследования. Генетический поиск может основываться на конкретных фенотипических признаках заболевания и данных семейного анамнеза. При их отсутствии предпочтительно использовать комплексную генетическую панель генов-кандидатов [49][50].

## Патологоанатомический диагноз

При патогистологическом исследовании с окраской гематоксилином и эозином, а также с использованием различных антител при ИГА, патологоанатомический диагноз формулируется в соответствии с классификацией нейроэндокринных опухолей гипофиза ВОЗ от 2022 г., который должен включать наличие нейроэндокринной опухоли гипофиза, ведущий фактор транскрипции и наименование клеток со степенью дифференцировки. Например, «высокодифференцированная нейроэндокринная опухоль гипофиза, состоящая из аденогипофизарных клеток PIT1-линии с соматотрофной (или маммосоматотрофной) дифференциацией». Оценка индекса пролиферации Ki-67 и нейроэндокринных маркеров (наиболее чувствительным считается синаптофизин) остается обязательной для всех типов и подтипов новообразований гипофиза [52].

Для гистологических подтипов PIT1-клеточного типа можно использовать диагноз «нейроэндокринная плотногранулированная (или редкогранулированная) соматотропная опухоль», «нейроэндокринная плотногранулированная (или редкогранулированная) лактотропная опухоль», «нейроэндокринная маммосоматотропная опухоль», «нейроэндокринная смешанная соматотропная и лактотропная опухоль», «нейроэндокринная опухоль из ацидофильных стволовых клеток», «нейроэндокринная тиреотропная опухоль», «нейроэндокринная зрелая полигормональная опухоль PIT1-линии», «нейроэндокринная незрелая опухоль PIT1-линии». Для диагностики новообразований данного типа и определения его подтипов экспертами ВОЗ рекомендовано использовать коммерческие антитела к ГР, пролактину, ТТГ, цитокератину, PIT1, ERα, реже GATA3, рецепторам соматостатина 2 подтипа.

Для опухолей TPIT-клеточного типа выставляется патологоанатомический диагноз «нейроэндокринная плотногранулированная (или редкогранулированная) кортикотропная опухоль» или «нейроэндокринная кортикотропная опухоль из клеток Крука». Для этого необходимо использовать антитела к АКТГ, цитокератину, TPIT, возможно к рецепторам соматостатина 5 подтипа.

Для опухолей SF1-клеточного типа выставляется патологоанатомический диагноз «нейроэндокринная гонадотропная опухоль». В панель антител входят SF1, ERα, при необходимости — GATA3, цитокератин, гонадотропины (ФСГ и ЛГ). При идентификации опухолей без определенного клеточного типа (нуль-клеточные или плюригормональные) применяют широкую панель антител ко всем гормонам аденогипофиза, факторам транскрипции и цитокератину. Нуль-клеточная опухоль является диагнозом исключения и требует тщательной дифференциальной диагностики с опухолями другого гистогенеза.

В Международной классификации болезней 11-го пересмотра (МКБ-11) ГипНЭО кодируется как 2D12.Y «Злокачественные новообразования других желез внутренней секреции или родственных структур» [53].

## Гормональное обследование

Рекомендуемый протокол типового гормонального обследования при опухолях гипофиза включает: пролактин, ГР, ИРФ-1, ТТГ, св.Т4, св.Т3, ЛГ, ФСГ, базальный кортизол, супрессивный тест с 1 мг дексаметазона, у пременопаузальных женщин — эстрадиол, у мужчин — общий тестостерон [6].

Рутинное применение иммунолокализации гипофизарных факторов транскрипции вместе с аденогипофизарными гормонами имеет решающее значение в эпоху прецизионной медицины. Ошибочный диагноз может привести к гораздо большим финансовым затратам, чем стоимость углубленного морфологического анализа. Следует подчеркнуть, что для качественного лечения опухолей гипофиза требуется опытная мультидисциплинарная команда, в которой патологоанатом является полноправным участником лечебного процесса, способствуя персонификации послеоперационного лечения с учетом результатов гистологического типирования.

## Обсуждение

Следует отметить, что переклассификация аденом гипофиза в нейроэндокринные опухоли вызвала раскол и дебаты среди международного экспертного сообщества эндокринологов. Ниже представлены основные возражения против целесообразности введения данной номенклатуры и «малигнизации» аденом гипофиза.

1. Предлагаемое номенклатурное изменение присваивает ярлык онкологии аденомам гипофиза, которые в подавляющем большинстве случаев являются доброкачественными и неинвазивными новообразованиями, и которые можно вылечить хирургическим путем или контролировать с помощью медикаментозной терапии. В отличие от НЭО, аденомы гипофиза имеют очень широкое распространение, но отличаются медленным течением и редко малигнизируются (1 случай на 100 000 аденом).

2. Отмечено, что такие биомаркеры, как синаптофизин, нейрон-специфическая энолаза, специфичны не только для нейроэндокринных клеток и также экспрессируются в новообразованиях щитовидной железы и надпочечников, что дает право считать аденомы надпочечников и узловые образования щитовидной железы также нейроэндокринными опухолями. Поскольку разница между эндокринной и нейроэндокринной клеткой строго не определена, то изменение названия, относящееся исключительно к гипофизу, без критического обзора общей таксономии эндокринных желез, сбивает с толку и запутывает классификацию эндокринных опухолей.

3. В настоящее время отсутствует корреляция между гистологическим диагнозом и клиническим поведением новообразований гипофиза, недоступны прогностические маркеры злокачественной прогрессии и ни один гистопатологический маркер надежно не предсказывает биологическое поведение опухоли гипофиза. Также отсутствуют корреляции между генетическими аномалиями и инвазивным или злокачественным опухолевым ростом. Отмечено, что многие негистологические факторы, например, радиологические признаки инвазивности или относительная интенсивность опухолевого сигнала на Т2-взвешенных МР-изображениях, также оказывают влияние на прогноз лечения аденом гипофиза и должны быть учтены в комплексной классификации.

4. Принимая во внимание низкую распространенность агрессивных аденом в популяции, произведенная смена терминов «аденома» на «опухоль» гипофиза вводит клиницистов в заблуждение и вызывает дополнительную тревогу у пациентов, поскольку придает всем аденомам гипофиза (даже с минимальным размером и доброкачественным течением) негативный прогноз, влияющий на качество жизни. Несмотря на то что в качестве переходного этапа новая редакция сохраняет возможность совместного использования двух терминов «ГипНЭО/гипофизарная аденома», это еще больше усложняет стратификацию заболевания и вызывает дополнительные проблемы в организации лечебной помощи и общении с медицинскими страховыми компаниями.

5. Не учтено мнение пациентов, их представителей, а также общественности, поскольку выбор адекватных терминов для описания поражений гипофиза особенно важен для пациентов и лиц, ухаживающих за ними. Такие ярлыки болезни, как рак, узел и опухоль, играют важную роль в восприятии риска, определении прогноза и принятии решения. Пациенты с низким риском прогрессирования заболевания с меньшей вероятностью получат пользу от лечения и с большей вероятностью испытают пагубные побочные эффекты вмешательств, тревогу, вызванную ярлыком болезни, и финансовый ущерб, возникающий в результате дополнительных и часто ненужных тестов и лечения. Не исключено возникновение различных психических, социальных, медицинских и экономических проблем.

6. Эксперты утверждают, что решение о смене номенклатуры было принято вопреки мнению многих специалистов. Согласно итоговому заключению междисциплинарного семинара гипофизарного общества на тему «Номенклатура новообразований гипофиза (PANOMEN)» от 2021 г., изменение терминологии не решает основной проблемы прогнозирования риска малигнизации, присваивает неподтвержденный диагноз злокачественности доброкачественным аденомам гипофиза и может неблагоприятно влиять на пациентов. В итоге 48 ведущих международных экспертов подписали статью «Выдержит ли аденома испытание временем?», в которой выразили серьезные опасения по поводу предлагаемого изменения дефиниции, рекомендовали сохранить термин «аденома» и вернуться к этой теме по мере появления новых данных о биологии новообразований гипофиза [2][21][52–56].

С другой стороны, адепты переименования представляют свои резоны, указывая, что точная верификация диагноза подразумевает не только идентификацию гистологического варианта опухоли, но и определение ее молекулярно-генетических и иммунологических характеристик, влияющих на клиническое течение и чувствительность к терапии. Соблюдение представленного протокола стратификации также позволяет идентифицировать эктопическую опухоль или метастатический очаг с неясным происхождением [57][58].

По мнению J. Trouillas и соавт., «клинически агрессивные» опухоли и карциномы статистически сходны по возрасту пациентов в дебюте заболевания, гендерным проявлениям, типу опухоли, величине пролиферативного индекса Ki-67 и экспрессии белка p53. Большинство гипофизарных карцином происходит из инвазивных макроаденом, являются резистентными к медикаментозной терапии и требуют повторных хирургических и радиологических вмешательств для достижения контроля. К сожалению, пока не существует верифицированных гистологических критериев для дифференциальной диагностики гипофизарной карциномы в первичных опухолях. Однако потенциальные признаки злокачественного поведения можно заподозрить на основании присутствия опухолевой инвазии, неоангиогенеза, сосудистой инвазии, патологических митозов, повышенных значений Ki-67 >10%, высокой иммунореактивности белка p53 и наличия геномных альтераций. Согласно результатам опроса европейского общества эндокринологов, локально агрессивные опухоли и карциномы имеют много общего и могут рассматриваться как «две стороны одной медали» [21][59].

Некоторые авторы поддерживают предложение использовать сочетанную дефиницию «аденома/ГипНЭО» в качестве переходной терминологии. Полагают, что переход на новую номенклатуру ГипНЭО потребует много времени для достижения социального консенсуса и признания [51, 60, 61].

В целом отношение эндокринологов к классификации от 2022 г. неоднозначное. С одной стороны, в новой редакции получил дальнейшее развитие цито- и гистогенетический подход при формировании патологоанатомического диагноза, что, безусловно, повысило его достоверность, особенно в случаях наличия эктопированной опухоли или метастатического очага. С другой стороны, столь кардинальная смена дефиниций с «аденомы» на «опухоль» и ожидаемые в связи с этим медицинские и психологические проблемы вызывают много вопросов. В первую очередь остается неясным, можно ли присваивать диагноз «ГипНЭО» любому пациенту с инциденталомой гипофиза или только послеоперационным больным с выставленным морфологическим диагнозом? Ведь существует масса новообразований гипофиза, не требующих хирургического вмешательства. Тогда какой диагноз ставить тем больным, которым, в силу разных причин, операция не проведена? Нельзя же выставить онкологический диагноз без цитологического анализа. И каким будет формат участия эндокринологов и онкологов в комплексном лечении больных с нейроэндокринными опухолями гипофиза? Все эти темы нуждаются в обсуждении.

В принципе, дебаты идут по поводу относительно небольшой клинической подгруппы аденом гипофиза, составляющей приблизительно 30% от всех опухолей и отличающейся высокой пролиферативностью, инвазивностью, плохой резектабельностью и частым рецидивированием [35]. Клетки данных опухолей характеризуются слабой видовой специализацией, активным пролиферативным потенциалом, несовершенным рецепторным фенотипом и низкой чувствительностью к влиянию модуляторов [13]. Как и все низкодифференцированные гормонально активные неоплазии, они обладают относительно меньшей секреторной активностью, компенсируемой высокой митотической способностью. В силу рецепторной несостоятельности эти опухоли отличаются рефрактерностью к супрессивной таргетной терапии и выпадают из общепринятой схемы лечения. Характерным примером являются редкогранулированные соматотрофные опухоли, резистентные к АС1 в силу низкой экспрессии 2-го подтипа СР и/или нарушения пострецепторных механизмов [7][62–64].

Врачей беспокоит не столько риск злокачественной трансформации, сколько тенденция агрессивных новообразований к продолженному росту и резистентность к адъювантному лечению. К сожалению, категория пациентов с агрессивным течением характеризуется запоздалой идентификацией, чему способствует недостаточная распространенность методов опухоль-ориентированной диагностики и существующая практика длительного подбора фармакотерапии методом «проб и ошибок». Поэтому чем скорее на основании клинических, инструментальных и морфологических маркеров будет выявлена «агрессивная» аденома, тем успешнее будет ее курабельность.

По сути, с учетом специфики происхождения клеток аденогипофиза и присутствия характерных цитонейромаркеров, речь действительно идет о наличии нейроэндокринных образований гипофиза. Поскольку основным возражением против данной классификации является неоправданное «озлокачествление» всех аденом, то почему бы в таком случае не присвоить клинически и морфологически доброкачественным образованиям малого размера диагноз «нейроэндокринная аденома», отделив их таким образом от подгруппы «инвазивных и рефрактерных» аденом, за которыми и закрепить термин «нейроэндокринная опухоль» (ГипНЭО)? Такое разделение способствовало бы снятию многих возражений и созданию мультидисциплинарных дифференциально-диагностических алгоритмов. К сожалению, клинико-патологическая классификация, определяющая прогноз, терапию и исходы всех аденом гипофиза с использованием современных методов клинико-лабораторного, радиологического и морфологического анализа, в настоящее время недоступна. Поэтому на современном этапе требуются дополнительные научные исследования. Актуальным является создание прогностических моделей для разделения аденом гипофиза на доброкачественные и агрессивные клинические подтипы с использованием современных диагностических возможностей.

## Заключение

Несмотря на то что новая классификации ВОЗ повлечет за собой необходимые организационные и методические изменения, ее клиническое воплощение будет способствовать повышению онкологической настороженности и привлечению внимания к группе пациентов с агрессивным течением заболевания. В любом случае, внедрение дифференцированного подхода при лечении различных морфологических вариантов ГипНЭО позволит оптимизировать вторичное медицинское пособие и уменьшить психологические, клинические и финансовые издержки неэффективного лечения. По мнению экспертного сообщества, не исключено, что в ближайшем будущем мы станем свидетелями новой комплексной классификации, объединяющей клиническую, генетическую, биохимическую, радиологическую, патологическую и молекулярную информацию для всех новообразований передней доли гипофиза [7][56]. Поскольку мнения ведущих международных экспертов радикально разделились, окончательное решение вопроса о целесообразности переименования будет зависеть от итоговых результатов дальнейшей дискуссии специалистов, закрепленных в международных рекомендациях.

## Дополнительная информация

Источники финансирования. Работа выполнена по инициативе авторов без привлечения финансирования.

Конфликт интересов. Авторы декларируют отсутствие явных и потенциальных конфликтов интересов, связанных с содержанием настоящей статьи.

Участие авторов. Пронин В.С. — концепция и дизайн исследования, сбор и анализ литературных данных, написание текста; Анциферов М.Б. — концепция и дизайн исследования, анализ представленных литературных данных; Алексеева Т.М. — дизайн исследования, сбор и обработка литературных данных; Пронин Е.В. — дизайн исследования, сбор и обработка литературных данных, написание текста; Лапшина А.М. — концепция и дизайн исследования, анализ литературных данных, написание текста; Урусова Л.С. — концепция и дизайн исследования, анализ литературных данных. Все авторы одобрили финальную версию статьи перед публикацией, выразили согласие нести ответственность за все аспекты работы, подразумевающую надлежащее изучение и решение вопросов, связанных с точностью или добросовестностью любой части работы.
